# State- versus trait- dependent immune alterations in major depression: Exploring CRP, numerical and functional changes in neutrophils and monocytes

**DOI:** 10.1192/j.eurpsy.2025.165

**Published:** 2025-08-26

**Authors:** J. Steiner

**Affiliations:** Psychiatry and Psychotherapy, University of Magdeburg, Magdeburg, Germany

## Abstract

**Background:**

Inflammatory processes and innate immune system activation have been implicated in psychiatric disorders. While our prior research highlighted elevated neutrophils, monocytes, and C-reactive protein (CRP) associated with symptom severity in schizophrenia, this study investigates whether similar immune alterations characterize major depression (MD).

**Methods:**

Differential blood counts, CRP levels, depression severity (HAMD-21), and psychosocial functioning (GAF) were assessed in controls (n = 129) and patients with first-episode (FEMD: n = 82) or recurrent (RMD: n = 47) MD at hospital admission (T0) and after 6 weeks of treatment (T6). Functional immune parameters, including the phagocytic activity of neutrophils and monocytes, were also measured in a subset of patients with MD (n = 16) and healthy controls (n = 27).

**Results:**

At T0, both FEMD and RMD patients exhibited increased neutrophils (p = 0.034) and CRP levels (FEMD: p < 0.001, RMD: p = 0.021) and decreased eosinophils (FEMD: p = 0.005, RMD: p = 0.004) compared with controls, adjusted for covariates (smoking, BMI, gender). Baseline lymphocyte counts were elevated in RMD (p = 0.003) but not FEMD. Functional analyses revealed significantly increased phagocytic activity of neutrophils in MDD patients compared to controls, both at baseline and T6. Changes in monocyte phagocytic activity correlated with ΔHAMD, indicating a link between immune cell function and symptom improvement.

At T6, eosinophils increased in FEMD (p = 0.011) without significant changes in RMD. Improvement in depression severity correlated with changes in neutrophil counts in FEMD (r = 0.364, p = 0.024). Comparatively, immune alterations in MD showed smaller effect sizes than those observed in schizophrenia. Notably, lymphocyte elevations were specific to recurrent MD, suggesting potential involvement of adaptive immunity in chronic MD.

**Conclusions:**

These findings highlight state- and trait-dependent immune alterations in MD, including heightened neutrophil activity in early stages and adaptive immune involvement in recurrent cases. Functional data further support the role of innate immune activation in MD, with phagocytic activity potentially serving as a biomarker for treatment response. Future studies may inform stage-specific immune-targeted interventions in MD.

**Disclosure of Interest:**

None Declared

